# Genetic diversity among *Plasmodium vivax* isolates along the Thai–Myanmar border of Thailand

**DOI:** 10.1186/s12936-016-1136-6

**Published:** 2016-02-09

**Authors:** Sarunya Maneerattanasak, Panita Gosi, Srivicha Krudsood, Jarinee Tongshoob, Charlotte A. Lanteri, Georges Snounou, Srisin Khusmith

**Affiliations:** Department of Microbiology and Immunology, Faculty of Tropical Medicine, Mahidol University, 420/6 Rajvithi Road, Bangkok, 10400 Thailand; Department of Immunology and Medicine, Armed Forces Research Institute of Medical Science-United States Army Military Component, Bangkok, Thailand; Clinical Malaria Research Unit, Faculty of Tropical Medicine, Mahidol University, Bangkok, Thailand; UPMC UMRS CR7, Sorbonne Universités, UPMC Univ Paris 06, 75005 Paris, France; Institut National de la Santé et de la Recherche Médicale (Inserm) U1135 – Centre National de la Recherche Scientifique (CNRS) ERL 8255, Centre d’Immunologie et de Maladies Infectieuses (CIMI) – Paris, 75013 Paris, France; Center for Emerging and Neglected Infectious Diseases, Mahidol University, Bangkok, Thailand

**Keywords:** *Plasmodium vivax*, Genetic diversity, Multiplicity, *Pvcsp*, *Pvmsp1*, *Pvmsp3α*, Thailand

## Abstract

**Background:**

Knowledge of the population genetics and transmission dynamics of *Plasmodium vivax* is crucial in predicting the emergence of drug resistance, relapse pattern and novel parasite phenotypes, all of which are relevant to the control of vivax infections. The aim of this study was to analyse changes in the genetic diversity of *P. vivax* genes from field isolates collected at different times along the Thai–Myanmar border.

**Methods:**

Two hundred and fifty-four *P. vivax* isolates collected during two periods 10 years apart along the Thai–Myanmar border were analysed. The parasites were genotyped by nested-PCR and PCR–RFLP targeting selected polymorphic loci of *Pvmsp1*, *Pvmsp3α* and *Pvcsp* genes.

**Results:**

The total number of distinguishable allelic variants observed for *Pvcsp*, *Pvmsp1*, and *Pvmsp3α* was 17, 7 and 3, respectively. High genetic diversity was observed for *Pvcsp* (*H*_*E*_ = 0.846) and *Pvmsp1* (*H*_*E*_ = 0.709). Of the 254 isolates, 4.3 and 14.6 % harboured mixed *Pvmsp1* and *Pvcsp* genotypes with a mean multiplicity of infection (MOI) of 1.06 and 1.15, respectively. The overall frequency of multiple genotypes was 16.9 %. When the frequencies of allelic variants of each gene during the two distinct periods were analysed, significant differences were noted for *Pvmsp1* (*P* = 0.018) and the *Pvcsp* (*P* = 0.033) allelic variants.

**Conclusion:**

Despite the low malaria transmission levels in Thailand, *P. vivax* population exhibit a relatively high degree of genetic diversity along the Thai–Myanmar border of Thailand, in particular for *Pvmsp1* and *Pvcsp*, with indication of geographic and temporal variation in frequencies for some variants. These results are of relevance to monitoring the emergence of drug resistance and to the elaboration of measures to control vivax malaria.

## Background

In Thailand, malaria is forest-related, with the highest prevalences recorded along regions bordering Myanmar, Laos and Cambodia. Forty-seven per cent of malaria cases in Thailand are due to *Plasmodium vivax* [1]. The control of *P. vivax* poses particular problems because of this parasite’s propensity to relapse. This is now compounded by the emergence of drug resistance; recently, one case of chloroquine-resistant vivax malaria has been reported in Thailand [2]. Approximately 6 % of Thai patients treated with a short acting anti-malarial drug followed by primaquine suffer a relapse of vivax malaria within 28 days [3]. To date, knowledge of the biology and epidemiology of *P. vivax* is relatively limited. Epidemiological and genetic studies of *P. vivax* population are essential for understanding the population dynamics and epidemiology of this parasite, and are relevant to monitoring responses to drug treatment.

Many polymorphic genetic markers are available for characterizing *P. vivax* populations [4–11]. Analyses of polymorphism and population diversity of *P. vivax* have focused on parasite molecules that are under selection by host immunity [12–15]. Most targeted polymorphic loci are found on single-copy *P. vivax* genes: *msp1* coding for merozoite surface proteins (MSP1) (*Pvmsp1)*, *msp3alpha* coding for another merozoite surface protein (*Pvmsp3α*), and *csp* coding for circumsporozoite protein (CSP)(*Pvcsp*). *Pvmsp1* gene has a mosaic structure, with seven conserved blocks and six variable blocks [16]. Among these blocks, the variable block 10, previously designated as F3 fragment, is highly polymorphic and as such is a suitable genetic marker for population studies [5, 7, 17]. *Pvmsp3α* is characterized by a distinct alanine-rich central region [18] with a high degree of polymorphisms [11, 19, 20]. The *Pvcsp* harbours a central immunodominant region with a variable number of tandem repeats represented by two major variant types, VK210 and VK247 [21, 22] with a worldwide distribution [23]. The three loci above are considered to be under selective immune pressure.

The current genetic data on *P. vivax* population lags behind those on *P. falciparum*. This study aims to assess the genetic diversity using *Pvmsp1*F3, *Pvmsp3α* and *Pvcsp* loci in *P. vivax* isolates collected along the Thai–Myanmar border, and to investigate whether variations in frequencies occur geographically and temporally. This data is intended to help understand the dynamics of *P. vivax* populations, and to support the design of effective control measures.

## Methods

### Samples

Two hundred and fifty-four stored anonymous frozen packed red cells collected during two periods: May 2003 to August 2004 (n = 173) and March 2012 to March 2013 (n = 81) were studied. DNA was extracted from the samples collected from patients presenting with *P. vivax* in Thai clinics along the Thai–Myanmar border in the west. The area is considered as having low and seasonal malaria transmission. The annual malaria incidence rates along Greater Mekong Subregion were 0.13–10.15 to 6/1000 populations in 2008 [24]. However, based on the record, some of the infections were probably acquired in the bordering countries. These isolates were from patients with the mean age (mean ± SD) of 25.28 ± 7.8 (range 14–57) and the mean % parasitaemia of 0.32 (range 0.1–1.19) during 2003–2004; and of 29.8 ± 11.5 (range 15–58) and the mean % parasitaemia of 0.401 (range 0.1–1.67) during 2012–2013. This cross sectional retrospective study was approved by the Ethics Committee of the Faculty of Tropical Medicine, Mahidol University (Approval number: MUTM 2013-034-01).

### DNA template preparation

*Plasmodium vivax* genomic DNA was extracted from 200 µL of frozen-packed red cells using the commercially available DNA Blood kit according to the manufacturer’s instructions (QIAGEN, Germany). The volume of the template obtained was 100 µL, thus each 1 µL corresponds to the DNA present in 2 µL of whole blood. Confirmation of the microscopic diagnosis examination was achieved by a multiplex real-time PCR assay using both genus- and species-specific primers [25].

### Genotyping of *Pvmsp1*F3 and *Pvmsp3α* by Nested PCR

Previously described primers for *Pvmsp1*F3 and *Pvmsp3α* [7] were used to amplify the polymorphic loci by nested PCR, with some modification. Briefly, amplification reactions were performed in a total volume of 25 µL containing 1X MyTag Red Mix (Bioline, UK) and 40 nM of each oligonucleotide primer. The amplification was initiated with 1 µL of the template genomic DNA, followed by the second amplification, in which 1 µL of diluted PCR products from the first amplification (at 1:1000 for *Pvmsp1*F3 and 1:100 for *Pvmsp3α*) were used. The cycling parameters were as follows: initial denaturation at 95 °C for 3 min; 30 cycles of denaturation at 95 °C for 1 min, followed by annealing at 60 °C for 1 min and extension at 72 °C for 1 min, and a final extension at 72 °C for 5 min. All PCR products were then analysed by electrophoresis on 3 % agarose gels and visualized under UV illumination after staining with ethidium bromide. The size of the amplified fragments was estimated by comparison to a 100 bp ladder marker. Each PCR product was screened for polymorphic banding by GeneTools analysis software (Syngene, UK). The genetic diversity of each marker was analysed by the number of alleles and the expected virtual heterozygosity (*H*_*E*_), The expected virtual heterozygosity (HE), defining as the probability that a randomly chosen pair of alleles differed from each other can range between 0 and 1, with values close to 1, reflecting high genetic diversity levels in a population [7]. The formula of expected heterozygosity (HE) is [n/(n − 1)] × (1 − ∑pi2), where “n” is the number of samples analysed and Pi is the proportion of the parasite population having allele i. The mean multiplicity of infection (MOI) was calculated by dividing the total number of *P. vivax* clones by the number of PCR-positive isolates [7].

### Genotyping of *Pvcsp* by PCR–RFLP

*Pvcsp* genotyping was based on restriction fragment length polymorphism analysis of the PCR products (PCR–RFLP) [5]. Briefly, *Pvcsp* was amplified by nested-PCR assay in a total of 25 µL and in presence of a reaction master mix containing 1X MyTag Red Mix (Bioline, UK) and 40 nM of each primer. One microlitres of template DNA was used to initiate amplification, followed by a second amplification, in which 1 µL of PCR products from the first amplification was used. The cycling conditions were as follows: 1 cycle of 95 °C for 5 min, 25 cycles (first amplification) and 30 cycles (second amplification) of denaturation at 94 °C for 1 min, followed by annealing at 62 °C for 2 min and extension at 72 °C for 2 min, and final extension at 72 °C for 5 min. Subsequently, 20 µL of the second PCR products were separately digested by restriction enzymes *Alu*I and *Bst*NI (New England Biolabs Inc., UK) according to the manufacture specification for 2 h in a total volume of 30 µL. The DNA fragments were separated by electrophoresis on 3 % agarose gel and visualized under UV illumination after ethidium bromide staining.

### Statistical analysis

Statistical analyses were performed using SPSS software version 15.0. The frequencies of allelic variants in each marker between two different periods of sample collection were analysed using Chi square or Fisher’s exact test as appropriate. The *P* value was estimated by Monte Carlo based on the number of 10,000 randomly sampled data sets. *P* value of < 0.05 were considered as statistically significant.

## Results

### Genetic diversity and multiplicity of *Pvmsp1*F3, *Pvmsp3α* and *Pvcsp*

**Fig. 1:**
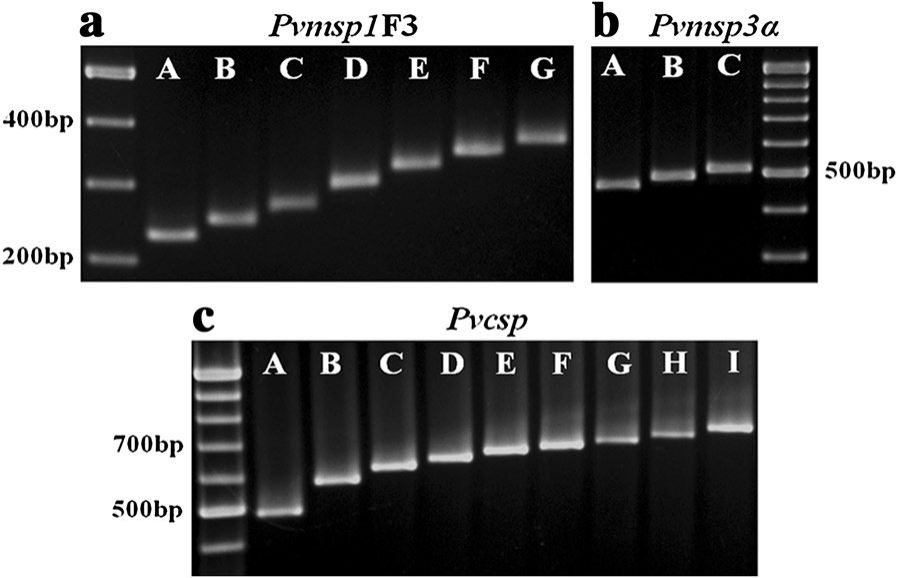
Fragment size ranges for *Pvmsp1*F3, *Pvmsp3α* and *Pvcsp* genes in *P. vivax* isolates from Thailand. Gel electrophoresis of PCR products: **a** *Pvmsp1*F3, **b** *Pvmsp3α* (B), **c** *Pvcsp*. The variants were coded alphabetically starting from the smallest size. A 100 bp ladder was used as molecular weight marker (M)

**Fig. 2:**
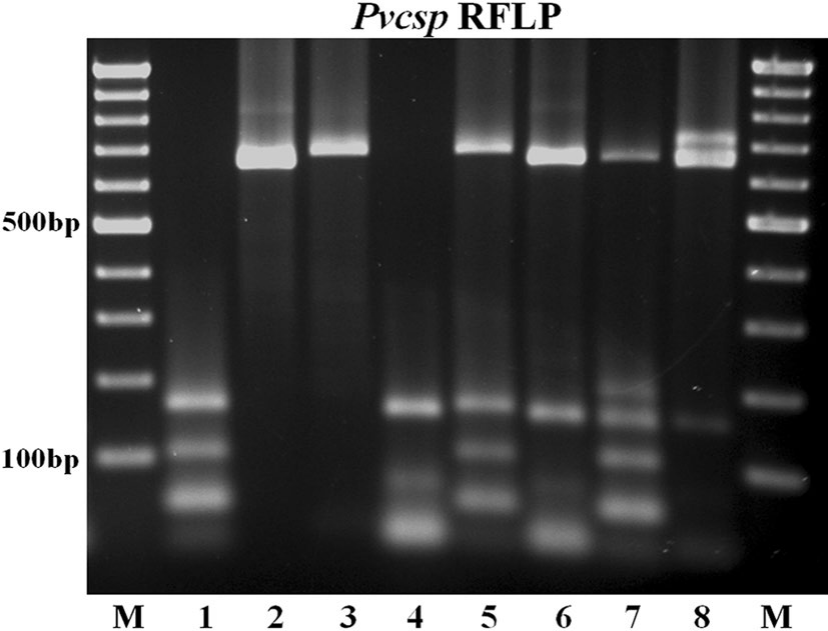
RFLP analysis of the *Pvcsp* fragments. In *lanes with odd numbers*, the fragments were digested with *Alu*I (an enzyme that cuts repeatedly in the VK210 repeat region). In *lanes with even numbers*, the fragments were digested with *Bst*NI (an enzyme that cuts repeatedly in the VK247 repeat region). Paired digestions from the fragments obtained from a single isolate are presented in *lanes 1–2* (a VK210 variant), *lanes 3–4* (a VK247 variant), and in *lanes 5–6* and *7–8* in which mixed genotypes are present. A 100 bp ladder was used as molecular weight marker (M)

**Fig. 3:**
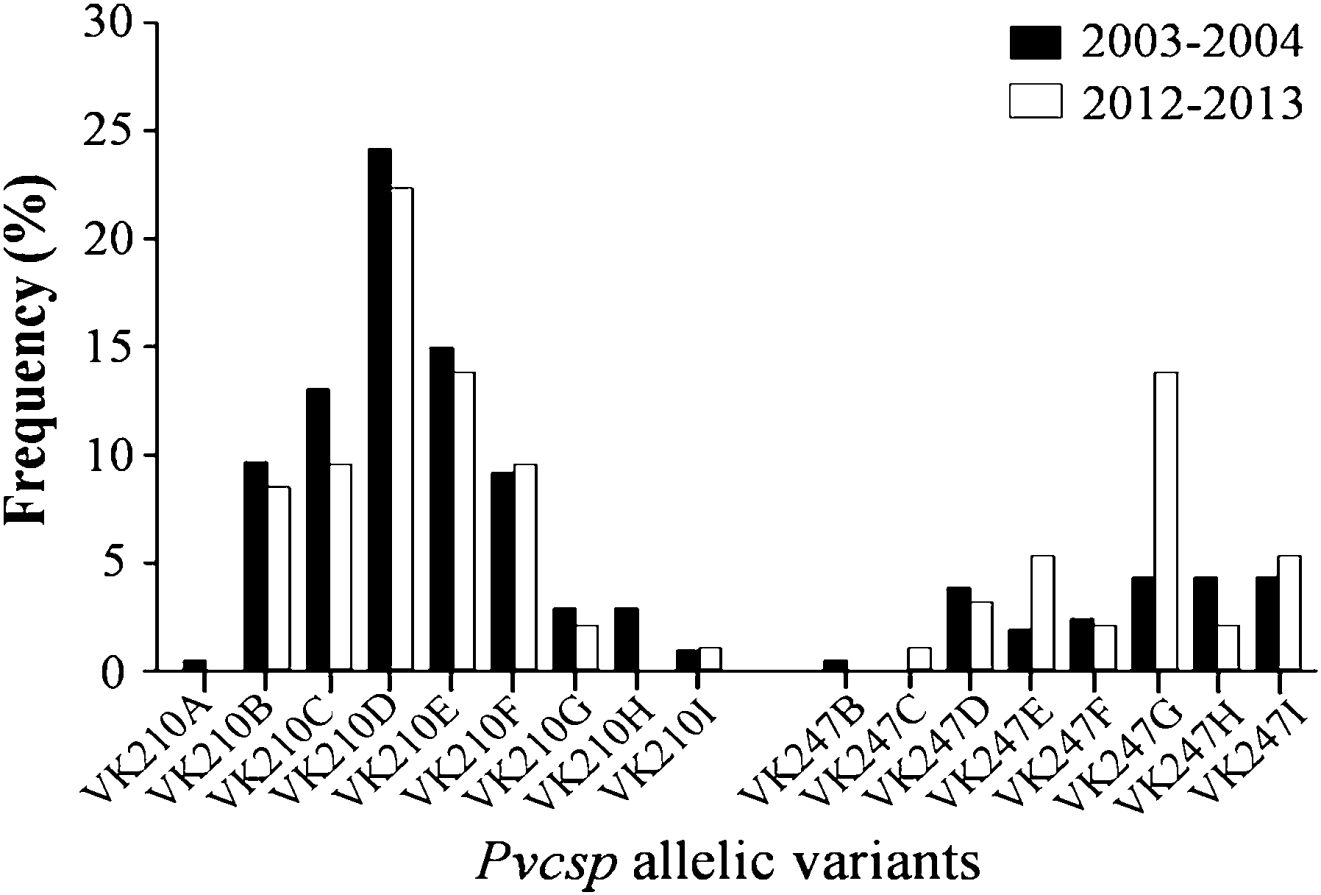
Frequency distribution of *Pvcsp* allelic variants from Thai *P. vivax* isolates collected over two periods. The number of isolates in the period of 2003–2004 (n = 173) and of 2012–2013 (n = 81) were analysed. Seventeen allelic variants were defined according to size (A to I) and repeat types (VK210 and VK247)

Amplification of the *Pvmsp1*F3 and *Pvcsp* fragments was achieved successfully for all the 254 *P. vivax* isolates, whereas the *Pvmsp3α* fragment could not be amplified in 45 isolates (18 %) despite repeated attempts (Table 1). The PCR fragments obtained were divided into bins that differed by 20 bp. Seven distinguishable *Pvmsp1*F3 size variants (designated A to G) with a size range of 220–360 bp (Fig. 1a) were observed with one variant (C, 251–270 bp) predominant at a frequency of 44 %. Three allelic size variants (designated A to C, 450–510 bp) were observed for the *Pvmsp3α* (Fig. 1b) at frequencies of 19, 34, and 47 %, respectively. Nine size variants (designated A to I, range 503–760 bp) were noted for *Pvcsp* (Fig. 1c) with the predominant variant (D, 641–660 bp) found at a frequency of 27 %. *Pvcsp* was further genotyped by RFLP [5] to distinguish between the two VK210 and VK247 repeat types (Fig. 2). When the PCR fragments are grouped by size and by repeat types, these could be divided into 17 different allelic forms, 9 PCR size variants for VK210 type and 8 for VK247 type (Fig. 3). The highest frequency was found for VK210D in both collection periods.

**Table 1:** Genetic diversity for *Pvmsp1*F3, *Pvmsp3α* and *Pvcsp* in the *P. vivax* isolates

	*Pvmsp1*F3	*Pvmsp3α*	*Pvcsp*
No. of alleles	7	3	9
No. of clones	269	209	292
No. of PCR positive isolates (%)	254 (100)	209 (82)	254 (100)
MOI^a^	1.06	1.00	1.15
*H*_*E*_^a^	0.709	0.631	0.846

^a^The multiplicity of infection (MOI) was calculated by dividing the total number of *P. vivax* clones by the number of PCR-positive isolates; *H*_*E*_, the expected virtual heterozygosity (the chance of drawing a pair of alleles differs from each other) using the following formula: *H*_*E*_ = [n/(n − 1)] × (1 − ∑p_i_^2^), where p_i_ is the frequency of the ith allele and n is the number of isolates analysed

The multiplicity of infection (MOI) and the expected virtual heterozygosity (*H*_*E*_) for *Pvmsp1*F3, *Pvmsp3α* and *Pvcsp* are shown in Table 1. High genetic diversity was observed in *Pvmsp1*F3 (*H*_*E*_ = 0.709) and *Pvcsp* (*H*_*E*_ = 0.846). Mixed genotype infections were observed in *Pvmsp1*F3 (n = 11, 7 MOI of 2 and 4 MOI of 3) and in *Pvcsp* (n = 37, 36 MOI of 2 and 1 MOI of 3) with a mean MOI of 1.06 and 1.15, respectively.

### Distribution of allelic variants among *P. vivax* isolates along Thai–Myanmar border at different periods

**Fig. 4:**
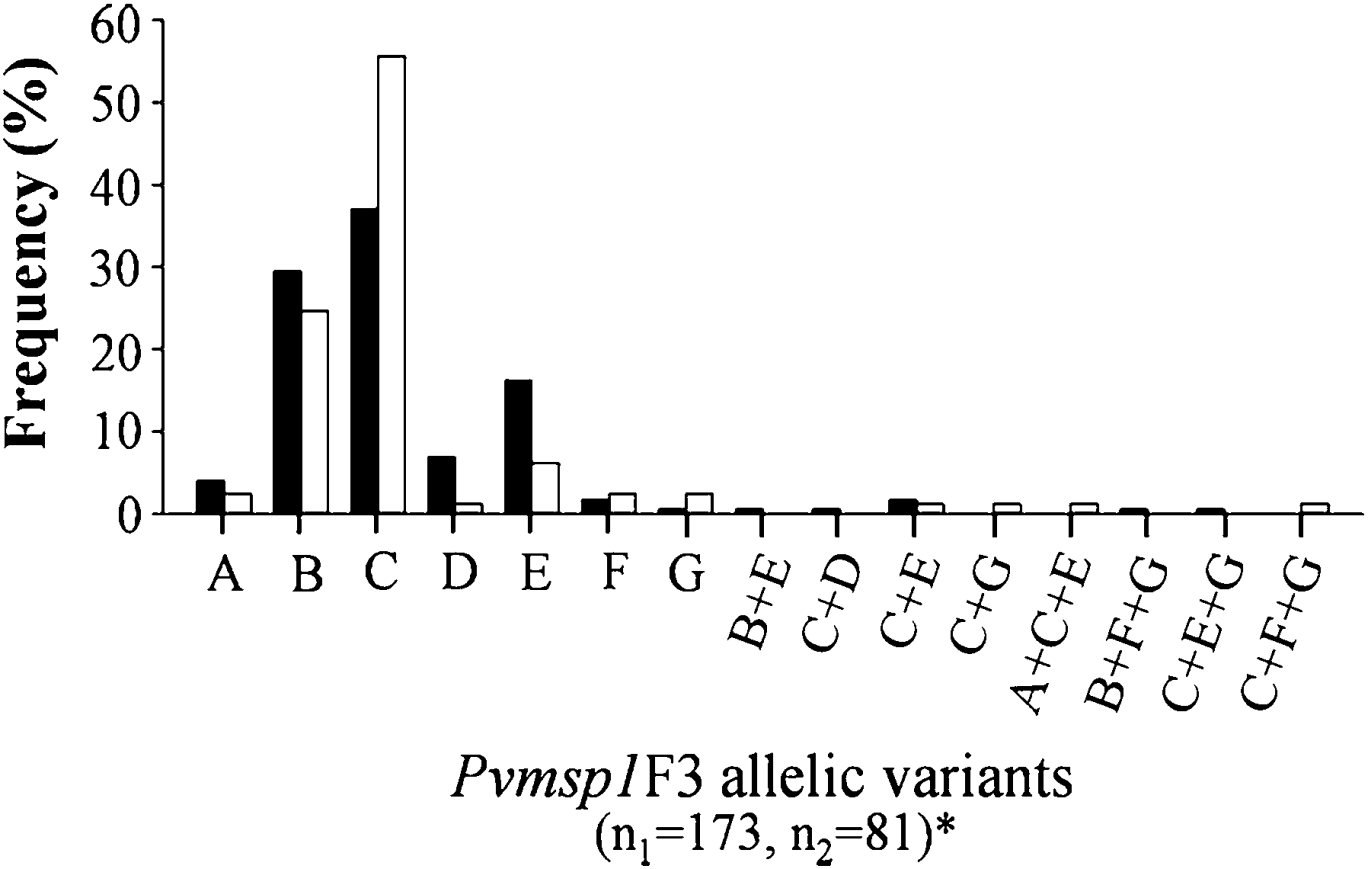
Distribution of *Pvmsp1*F3 allelic variants in *P. vivax* isolates collected along the Thai–Myanmar border during two periods. Distribution in 2003–2004 (*black bars*, n_1_ = number of isolates) and 2012–2013 (*white bars*, n_2_ = number of isolates) were shown. The allelic variants were designated A to G. Significant differences in allelic variant frequency between the two collection periods are indicated by *asterisk* (Fisher’s exact test, *P* = 0.018)

The *Pvmsp1*F3 genotypes found in *P. vivax* isolates collected over two periods from the Thai–Myanmar border were compared (Fig. 4). Seven allelic *Pvmsp1*F3 variants were observed with low frequencies of the A and G variants. Infection with the C variant was predominant among isolates (44 %), and for both collection periods that were separated by ten years (38 % during 2002–2003 and 56 % during 2012–2013). A significant difference in the frequencies of the *Pvmsp1*F3 genotypes was found between the two collection periods (Fisher’s exact test, *P* = 0.018).

**Fig. 5:**
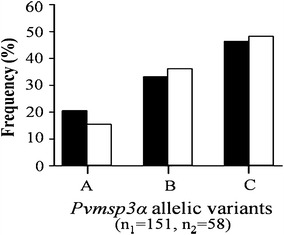
Distribution of *Pvmsp3α* allelic variants in *P. vivax* isolates collected along the Thai–Myanmar border during two periods. Distribution in 2003–2004 (*black bars*, n_1_ = number of isolates) and 2012–2013 (*white bars*, n_2_ = number of isolates) were shown. The allelic variants were designated A to C. Chi square test did not detect any significant difference in allelic variant frequencies between the two collection periods (*X* ^2^ = 0.701, degrees of freedom (df) = 2, *P* = 0.724)

All three *Pvmsp3α* allelic variants were observed in the isolates from this area (Fig. 5). Infections with the C variant were predominant among the isolates (47 %), and for both collection periods (46 % for 2002–2003, and 48 % for 2012–2013). No significant differences in *Pvmsp3α* genotype frequencies were noted for the isolates collected during the two collection periods (*X*^2^ = 0.701, degrees of freedom (df) = 2, *P* = 0.724).

**Fig. 6:**
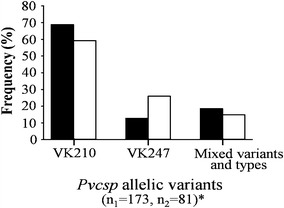
Distribution of single and mixed genotype infections of the *Pvcsp* repeat types (VK210 and VK247) in *P. vivax* isolates collected along the Thai–Myanmar border. Distribution in the period of 2003–2004 (*black bars*, n_1_ = number of isolates) and of 2012–2013 (*white bars*, n_2_ = number of isolates) were shown. Significant differences in allelic variant frequencies between the two collection periods are indicated by *asterisk* (*X* ^2^ = 6.879, degrees of freedom (df) = 2, *P* = 0.033)

All 17 *Pvcsp* allelic variants (9 VK210 and 8 VK247) were found among the isolates collected. The distribution of single and mixed genotype infections of *Pvcsp* repeat types (VK210 and VK247) during two periods of 10 years different are shown in Fig. 6. Mixed *Pvcsp* genotype infections, either different allelic variants or different *Pvcsp* repeat types, were found among isolates with similar frequencies in both periods of sample collection (19 % during 2003–2004; 15 % during 2012–2013). VK210-only infections were predominant among isolates (66 %), and for both collection periods (69 % for 2002–2003, and 59 % for 2012–2013). Significant difference in frequencies of single and mixed *Pvcsp* genotype infections between the two periods was found (*X*^2^ = 6.879, degree of freedom (df) = 2, *P* = 0.033). The frequency of VK210-single infection was slightly lower, but VK247-single infection was higher among isolates during 2012-2013 (VK210, 59 %; VK247, 26 %) than those during 2003–2004 (VK210, 69 %; VK247, 13 %).

## Discussion

It has long been known that the morphologically similar parasites of a *Plasmodium* species are in fact biologically and immunologically highly diverse. Until the advent of molecular genotyping through amplification, the study of this diversity for the parasites of humans was limited to experimental infections, enzyme typing, differential reactivity to specific antibodies, and, for *P. falciparum*, to in vitro cultured material. The advent of molecular genotyping based on genetic amplification has not only widened investigations to large numbers of field samples, but also to the all parasite species, including the hitherto neglected *P. vivax*.

The purpose of this study was to assess the genetic diversity of *P. vivax* collected from patients presenting in the Thai–Myanmar border of Thailand, for which a collection of samples obtained 10 years apart were available and based on the increasing evidence of drug resistance in this border area by the report in 2002–2003 of chloroquine resistance in *P. vivax* in Dawei (Mon State), Myanmar [26]. Given that *P. vivax* infections are generally treated with chloroquine, an emergence in 2008 of *P. vivax* resistance to chloroquine in this border area was reported and their spread has probably contributed to maintain a higher than expected level of genetic diversity [2].

Three polymorphic loci commonly used for the study of *P. vivax* were used for this study: the repeat region of *Pvcsp*, the F3 fragment of *Pvmsp1*F3 and a segment of *Pvmsp3α*, which were evaluated by the number of distinguishable size allelic variants found in *P. vivax* isolates. The data obtained for the *Pvmsp1*F3 and *Pvcsp* loci revealed that the parasites displayed a degree of genetic diversity with a high proportion of mixed genotype infections, which are in line with previous findings on the genetic diversity of *P. vivax* isolates collected from diverse endemic areas of Thailand [5, 9]. Recently, by using different sets of highly polymorphic microsatellite markers, a number of studies of genetic variability and transmission dynamics of *P. vivax* isolates from different malaria endemic areas have been observed with similar degree of diversity in Brazil (HE = 0.71) (*H*_*E*_ = 0.71) [27]; Sri Lanka (*H*_*E*_ = 0.86)/Myanmar (*H*_*E*_ = 0.85)/Ethiopia (*H*_*E*_ = 0.75) [28], and Vietnam (*H*_*E*_ = 0.85) [29]. Moreover, some differences in the frequencies of *Pvmsp1*F3 variants and *Pvcsp* repeat types were noted among isolates in this border collected over the two distinct sample collection periods. Parasites with the *Pvcsp* VK210 repeat type dominated in all the samples sets analysed, a feature noted in other studies of isolates collected in Thailand [5, 9], Myanmar (98 %) [30], India (99 %) [6], Guyana (92 %) [31], Pakistan (96 %) and Iran (69 %) [8]. In the present study, VK247-single infection was higher among isolates collected during 2012–2013 (25 %) than those during 2003–2004 (13 %), with obviously higher in VK247G during 2012–2013 (14 %). This striking increasing incidence might cause by several selective constraints imposed by drug usage [32], as well as local vectors [33]. It is well documented from previous studies in different drug susceptibility between *P. vivax* VK247 subtype and VK210 subtype, in which the mean time to clear parasite DNA, as determined by quantitative PCR of *P. vivax* genotype-specific DNA during a treatment course, was significantly slower for VK210 parasites than for VK247 parasites [32]. Likewise, the differential susceptibility of mosquito vectors to *P. vivax* circumsporozoite phenotypes in southern Mexico was found, in which *Anopheles albimanus* is more susceptible to VK210 carrying parasites while *Anopheles pseudopunctipennis* is more susceptible to VK247 carrying parasites [33].

The *Pvmsp3α* fragment amplified in this study was relatively uninformative in that only three allelic variants were detected. In a previous study of *P. vivax* from Papua New Guinea employing the same amplification protocol, a high degree of diversity was observed, with 15 distinct allelic variants detected in 106 samples [7]. The contrast with the low diversity observed in this study is probably due to use of classical gel electrophoresis with a relatively low resolution (bin sizes of 20 bp) rather than capillary electrophoresis (bin sizes of 3 bp). This has also probably contributed to underestimate the diversity of *Pvmsp1*F3, since 28 distinct allelic variants were observed using capillary electrophoresis for the *P. vivax* isolates from Papua New Guinea [7]. It should be noted that genetic diversity is likely to be higher in Papua New Guinea where the malaria endemicity is higher than that on the Thai borders. Nonetheless, comparison of parasite genotyping data from different studies should be interpreted in the light of the resolution of the method used to distinguish between allelic variants. Sequencing of the allelic variants present in each would provide the most accurate data for genetic diversity studies; however, cost considerations preclude this for a number of researchers in endemic areas. Moreover, data from the standard protocols that are accessible to most is generally sufficient to provide a clear broad picture of parasite genetic diversity and to decide whether further more detailed studies would be warranted.

Whereas analyses of *P. falciparum* populations indicate that the MOI is broadly correlated with transmission levels [34–36], the observed MOI values for the Thai *P. vivax* isolates presented here were high despite the low levels of malaria transmission. This might indicate that the transmission levels are underestimated. It is probable that the presence of hypnozoites, which are the liver dormant forms that can activate at various times in the months and years following the infectious bite to initiate a blood infection, contributes significantly to this high level of diversity. *P. vivax* strains in Southeast Asia region are predicted to have a high relapse incidence (836 relapses per 100,000 person days) and a rapid mean time from primary episode to first relapse (41 days) [37]. Furthermore, analyses of relapse parasites clearly indicated that they are genetically diverse and often distinct from the parasites that appear during the primary infection [38, 39]. Another potential explanation is the geographical location of the site where the survey presented here was conducted. The proximity to the border of Myanmar where malaria endemicity is higher than in Thailand has probably contributed to increase diversity through the frequent movement of populations to and from Thailand, resulting in a steady introduction of strains. In the course of this study, a few *P. vivax* isolates were also collected at the Thai–Thai–Lao border (n = 1 during 2003–2004, and n = 10 during 2012–2013) and the Thai–Cambodia border (n = 3 during 2003–2004, and n = 10 during 2012–2013). Despite the limited number of isolates, they were found to harbour numerous distinct allelic variants for the three markers (five variants for *Pvmsp1F3*, threevariants for *Pvmsp3α*, and seven variants for *Pvcsp*, and three isolates had mixed *Pvcsp* genotype infections for the Thai–Lao boder; and six variants for *Pvmsp1F3*, three variants for *Pvmsp3α*, and 10 variants for *Pvcsp* and four isolates had mixed genotype infections at the Thai–Cambodia border). This might be due to the fact that *P. vivax* infections are generally treated with chloroquine, therefore, the emergence in 2008 of *P.vivax* resistance to chloroquine in areas of Myanmar bordering Thailand [26], and their spread has probably contributed to maintain a higher than expected level of genetic diversity [2].

Multiple genotype infections are common in malaria, and higher multiplicities of infections are likely to facilitate genetic recombination of parasites leading to the generation of novel strains [40], and could be an indicator of immune status [41]. Previous studies indicated that allelic recombination events are associated with the genetic diversity of *Pvmsp1*F3 [8, 16], *Pvmsp3α* [11, 20] *and Pvcsp* [10]. Mixed genotype *Pvmsp1*F3 or *Pvcsp* infections were found in 17 % of the isolates, a frequency similar to that observed in previous surveys in Thailand [5, 9], in India using *Pvmsp3α* [6], in Papua New Guinea using *Pvmsp1*F3 [42], or in Pakistan and Iran using *Pvmsp1* [8]. Knowledge of the genetic diversity can be used to assess geographical differentiation and explain the spatial distribution of alleles at a given loci, and provide a baseline information for surveillance systems directed to identifying the origin of isolates in areas with occasional imported malaria cases [40].

The extensive polymorphism that characterize the loci targeted in this study are considered to be the result of selective constraints to which *P. vivax* is subjected [43], which include immune responses in the vertebrate host [39], drug pressure [32], as well as vector competence [33]. In this study, the degree of diversity recorded for the three markers did not vary between two collection that were separated by 10 years, though the frequencies of some *Pvmsp1*F3 and *Pvcsp* allelic variants altered significantly with time. Given the relatively limited number of isolates analysed and for each period, it is unclear whether these variations are sotchastic or due to selective pressure.

## Conclusion

In summary, this study demonstrated that high genetic diversity and multiplicity of infection levels occur in the *P. vivax* parasites circulating along the Thai–Myanmar border despite the low level of malaria transmission to which the resident populations are subjected. The genotyping data presented will be useful to the selection of markers suitable for use in drug efficacy studies, and to an improved understanding of the epidemiology of *P. vivax* in Thailand, which can guide the design of measures to control and eventually eliminate this parasite.

## Abbreviations

*Pvcsp*: circumsporozoite surface protein gene of *Plasmodium vivax*
*Pvmsp1*: merozoite surface protein 1 gene of *Plasmodium vivax*
*Pvmsp3*α: merozoite surface protein 3 alpha gene of *Plasmodium vivax*
PCR: polymerase chain reaction
RFLP: restriction fragment length polymorphism
bp: base pair
MOI: multiplicity of infection
*H*_*E*_: expected virtual heterozygosity
